# Analysis of the p53 gene by PCR-SSCP in ten cases of Wilms' tumor

**DOI:** 10.1590/S1516-31802000000200005

**Published:** 2000-03-02

**Authors:** Ricardo Defavery, José Alexandre Rodrigues Lemos, Simone Kashima, José Eduardo Bernardes, Carlos Alberto Scridelli, Dimas Tadeu Covas, Luiz Gonzaga Tone

**Keywords:** Wilms' tumor, p53 gene, PCR-SSCP, Solid tumors, Tumor de Wilms, Gene p53, PCR-SSCP, Tumores sólidos

## Abstract

**CONTEXT:**

Mutations of the p53 tumor suppressor gene are the most frequent alterations observed in human neoplasias affecting adults. In pediatric oncology, however, they have seldom been identified. Wilms' tumor is a renal neoplasia commonly occurring in children and is associated with mutations of the WT1 gene. The correlation between Wilms' tumor and alterations of the p53 gene has not been well established, with a low frequency of mutations having been reported in this type of tumor. Mutation may be associated with advanced stage disease and unfavorable histology.

**OBJECTIVE:**

To screen for mutations of the p53 gene by the PCR- SSCP method and DNA sequencing in cases of Wilms' tumor suggestive of mutation.

**DESIGN:**

Case Report.

**CASE REPORT:**

Evaluations of exons 5-9 of the p53 gene in DNA samples extracted by PCR-SSCP from 10 Wilms' tumors in children at different stages, and DNA sequencing. Changes in SSCP analysis were observed in exon 8 in two samples. The probable mutations were not confirmed by DNA sequencing. The absence of point mutations in p53 gene observed in the 10 samples of Wilms' tumor studied agrees with literature data, with DNA sequencing being of fundamental importance for the confirmation of possible mutations.

## resumo

**CONTEXTO:**

Mutações do gene supressor tumoral p53 são observadas como as mais freqüentes alterações em neoplasias humanas em adultos. Em oncologia pediátrica, entretanto, são pouco identificadas. O tumor de Wilms é uma neoplasia renal comum em crianças e associa-se com mutações do gene WT1. A correlação entre tumor de Wilms e alterações do gene p53 não está bem estabelecida, sendo baixa a freqüência tumoral, podendo haver associação com doença em estágio avançado e histologia desfavorável.

**TIPO DE ESTUDO:**

Relato de caso.

**RELATO DE CASO:**

Foi realizar uma triagem para mutações do gene p53 pelo método de PCR-SSCP e seqüenciamento de DNA nos casos sugestivos de mutação em tumor de Wilms. Avaliação do gene p53 nos exons 5-9 em amostras de DNA extraídos de 10 tumores de Wilms de crianças em diferentes estágios pelo método de PCR-SSCP e seqüenciamento de DNA. Alterações na análise do SSCP foram observadas em duas amostras no exon 8. As prováveis mutações não foram confirmadas pelo seqüenciamento de DNA. A ausência de mutações de ponto do gene p53 observada nas 10 amostras de tumor de Wilms estudadas está de acordo com a literatura, sendo o seqüenciamento de DNA fundamental para a confirmação de possíveis mutações.

## INTRODUCTION

The p53 tumor suppressor gene is located on the short arm of human chromosome 17 (17p13) and it codes for a nuclear phosphoprotein of 53 kDa. The function of wild type p53 is the negative regulation of cell proliferation, with a transcription action that inhibits the G1 phase of the cell cycle in the presence of damaged DNA.^1-3^ p53 is the most intensely investigated gene in human cancer.^2^ Its mutations have been identified in 50% of adult neoplasias involving the colon, lung, esophagus, stomach, liver, breast, and uterine cervix.^4^ However, mutations of the p53 gene have been little observed in children.^5,6^

Wilms' tumor is the renal neoplasia most frequently occurring in children, with an incidence of 1:10,000 children, especially among those younger than 6 years.^7^ Approximately 5 to 10% of children with Wilms' tumor have bilateral involvement. Wilms' tumor is associated with congenital abnormalities including genitourinary malformations, sporadic aniridia, mental retardation, and hemihypertrophy.^8^

Mutations of the WT1 gene are associated with Wilms' tumor.^9^ The correlation between p53 gene and Wilms' tumor is not completely understood. There are few reports on mutations of this gene in this tumor type. A possible association with unfavorable prognosis and advanced stage of the disease has been reported.^6,9,10^

In the present study we investigated possible mutation in the regions of the 5-9 exons of the p53 gene in children with Wilms' tumor by the polymerase chain reaction – single strand conformational polymorphism (PCR-SSCP). Samples that presented anomalous migration by PCR-SSCP analysis were submitted to automatic DNA sequencing to confirm possible mutations.

## CASE REPORT

Tumor samples from 10 patients with a diagnosis of Wilms' tumor were studied by PCR-SSCP to determine the presence of possible mutations due to genetic changes in the p53 gene. The patients were seen at the Pediatric Oncology Outpatient Clinic of the University Hospital, Faculty of Medicine of Ribeirão Preto, University of São Paulo. The clinical and histological characteristics of the patients (7 girls and 3 boys) are presented in Table 1. Mean age at diagnosis was 4.1 years. Histology was favorable in 6 cases and unfavorable in 3. In one case of bilateral Wilms' tumor the material was insufficient for histopathological analysis. The tumor was stage I in 5 cases, stage III in 1, stage IV in 3, and stage V in 1. The classification of Wilms' tumor was based on the criteria of the National Wilms' Tumor Study.^11^ Tumor samples were obtained during surgery and DNA was extracted by the phenol/chloroform method.^12^

**Table 1:** Clinical and laboratory characteristics of patients with Wilms' tumor submitted to analysis of the p53 gene

Case	Age	Sex	Histology/Staging	Bilateral Metastases	Treatment	Clinical Outcome
1	1	F	* stage V	yes / no	act/ dox vin/ rad	DF (5 years)
2	3.7	F	favorable / stage I	no / no	act/vin	DF (2 years)
3	1.9	F	favorable / stage I	no / no	act/vin	DF (2 years)
4	5	M	favorable / stage III	no / abdominal	act/dox/vin/ cisp/eto/rad	death
5	1.8	M	favorable / stage I	no / no	act/vin	DF (4 years)
6	2	M	favorable / stage I	no / no	act/vin	DF (5 years)
7	5.2	F	favorable / stage IV	no / pulmonary	act/vin/ dox/rad	DF (3 years)
8	5	F	unfavorable / stage IV	no / pulmonary	act/vin/dox/ rad/cisp/ eto/ifo	death
9	12	F	unfavorable / stage IV	no / liver	cisp/eto/ifo	NI
10	4	F	favorable stage I	no / no	act/vin	DF (2 years)

act: actinomycin; dox: doxorubicin; vin: vincristine; cis: cisplatin; eto: etoposide; ifo: ifosfamide; rad: radiotherapy;

* preoperative chemotherapy; NI: no information; DF: disease free

The possible alterations of the p53 gene in the regions corresponding to exons 5-9 were evaluated by PCR- SSCP. Each of the five regions analyzed was amplified by PCR using a pair of corresponding primers^13^ (Table 2). For PCR, each DNA sample (0.1 mg/ml) was added to a mixture containing 2.5 mM buffer solution (0.2 M Tris- HCI, 0.5 M KCl, pH 8,4); 10 mM dNTPs (dATP, dCTP, dGTP, dTTP); 1.5 mM Mg for exons 5, 6, 7 and 8; 2 mM Mg for exon; a pair of primers (10 mg/ml) corresponding to the exon under study, and taq polymerase (5 U/ml), to a final volume of 25 ml. All samples were submitted to the following amplification conditions: 35 successive cycles of denaturation (1 minute at 94°C for all exons), annealing (1.5 minutes at 61°C for exons 5 and 8, 54°C for exon 6, 58°C for exon 7 and 53°C for exon 9) and extension (1 minute at 72°C for all exons).

**Table 2:** Primers, characteristics and amplified regions of gene p53 used in the present study

Name	Sequence	Sense antisense	Exon	Exon (bp)	Sequence (bp)
MH22	5'CT5GTTCACTTGTGCCCTGAC-3	Sense	5	184	274
MH20	5'-CAACCAGCCCTGTCGTCTCT-3	Antisense	5		
MH28	5'-GAGACGACAGGGCTGGTT-3'	Sense	6	113	230
MH29	5'-CCACTGACAACCACCCTT-3'	Antisense	6		
MH30	5'-CCAAGGCGCACTGGCCTC-3'	Sense	7	110	211
MH31	5'-GAGGCAAGCAGAGGCTGG-3'	Antisense	7		
MH19	5'-GGGACAGGTAGGACCTGATT-3'	Sense	8	137	223
MH23	5'-CACCGCTTCTTGTCCTGCTT-3'	Antisense	8		
MH34	5'TTATGCCTAGATTCACTTTTT-3'	Sense	9	74	163
MH25	5'-CATCGAATTCTGGAAACTTTCCACTTGAT-3'	Antisense	9		

bp: base pair; Exon 8

For SSCP, the PCR products were diluted 1:10 in a solution containing 0.1% SDS and 10 mM EDTA, and an equal volume of dye with 20 mM EDTA, 95% formamide, 0.05% bromophenol blue and 0.05% xylene cyanol was added. Denaturation was then carried out at 96°C for 10 minutes. A fraction of this solution was put into 6% nondenaturing polyacrylamide gel and submitted to electrophoresis under the following conditions: 8 W, 40 mA and 200 V for 2-4 hours at 4°C. The gel was stained with silver nitrate, developed, photographed, and analyzed.

For DNA sequencing of the samples with probable mutations screened by PCR-SSCP, a 100 ml aliquot of each PCR product was used for DNA purification. The purified DNA products and the primers labeled with fluorescein^13^ (Table 2) were submitted to the sequencing reaction using the Thermo Sequenase kit (Amersham Pharmacia, Little Chalfont, Buckinghamshire, UK) according to the manufacturer's instructions. Electrophoresis was then carried out using a 7% non-denaturing gel with an automatic ALF sequencer (Amersham Pharmacia Biotech, Uppsala, Sweden) at 1500 V, 25 mA and 60 W for 4-6 hours. The DNA sequences obtained for each sample were compared to the normal sequence of the p53 gene obtained from the Gene Bank, sequencing access number x54156.

The DNA samples from the 10 Wilms' tumor specimens were submitted to screening by PCR-SSCP for possible mutations of the p53 gene in the regions corresponding to exons 5-9.

**Figure 1:**
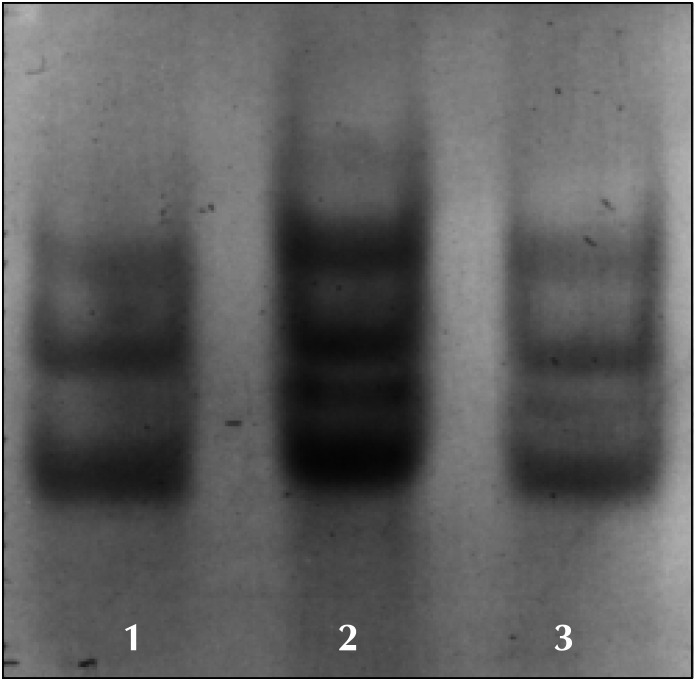
SSCP on exon 8. Numbers 1 and 3: Normal controls. Number 2: anomalous run (case 1).

Abnormal bands were revealed by SSCP in exon 8 in 2 samples. One was from a patient with bilateral involvement (case 1) (Figure 1) and the other from a patient with favorable histology (case 3). The clinical outcome was good for all of these patients. The DNA sequencing analyses of these 2 samples with altered migration in exon 8 were normal. Thus, point mutation alterations were not confirmed in the sequencing of DNA from the 2 samples.

## DISCUSSION

In the present study we assessed the presence of possible mutations due to genetic alteration in the regions between exons 5 and 9 of the p53 gene in 10 cases of Wilms' tumors affecting children. In 2 cases, altered electrophoretic migration was observed by SSCP but was not confirmed by DNA sequencing.

For the screening of samples with probable mutations of the p53 gene we used PCR-SSCP, a method with approximately 90% sensitivity and specificity for the detection of mutations confirmed by DNA sequencing in PCR products with 200 base pairs or less.^14,15^ Even though the coding region of the p53 gene consist of 10 exons (the first is non-coding), the analysis of mutations was performed in the region between exons 5 and 9. The reason for this is that more than 98% of the mutations of the p53 gene in human neoplasias are located in these exons.^5^ In addition, the region between exons 5 and 8 (codons 126 and 331 with 540 base pairs) contains DNA sequences that code for evolutionarily conserved domains considered to be functionally important.^3^

The false-positive results obtained here by SSCP in 2 cases agree with reports by other investigators who have studied the p53 gene in Wilms' tumor, such as Waber et al.^16^ and Malkin et al.,^9^ demonstrating that DNA sequencing is of fundamental importance in the determination of the existence of mutations in a given DNA segment.

According to several reports, the incidence of point mutations of the p53 gene in Wilms' tumors is small.

Kusafuka et al.^6^ studied 13 cases of Wilms' tumor and detected no mutation of the p53 gene. In a study of 38 cases of Wilms' tumor, Waber et al.^16^ also did not detect any mutation of the p53 gene. Malkin et al.^9^ detected 2 cases with mutations out of a total of 21. One of the patients had an advanced stage tumor with favorable histology and the other had focal anaplasia. Bardeesy et al.^10^ did not observe mutations of the p53 gene in 92 cases of Wilms' tumor with favorable histology.

Based on these findings, the absence of detection of mutations observed in the present study agrees with current data, despite the small number of cases analyzed. We suggest that the number of Wilms' tumor specimens to be evaluated should be increased in future studies, especially those from patients with unfavorable histology and advanced stage disease, and that more comprehensive studies should be performed by analyzing the remaining coding exons of the p53 gene and their expression, in order to better determine any possible association between alterations of p53 gene and Wilms' tumor.
